# Development of a Continuous Pulsed Electric Field (PEF) Vortex-Flow Chamber for Improved Treatment Homogeneity Based on Hydrodynamic Optimization

**DOI:** 10.3389/fbioe.2020.00340

**Published:** 2020-04-30

**Authors:** Felix Schottroff, Justus Knappert, Pauline Eppmann, Anna Krottenthaler, Tobias Horneber, Christopher McHardy, Cornelia Rauh, Henry Jaeger

**Affiliations:** ^1^Institute of Food Technology, Department of Food Science and Technology, University of Natural Resources and Life Sciences (BOKU), Vienna, Austria; ^2^Institute of Food Technology and Food Chemistry, Department of Food Biotechnology and Food Process Engineering, Technische Universität Berlin, Berlin, Germany

**Keywords:** pulsed electric fields (PEF), treatment chamber design, proof of concept, process optimization, numerical simulation, differentiation of thermal and electric field effects, non-thermal inactivation of microorganisms, effects of PEF on alkaline phosphatase

## Abstract

Pulsed electric fields (PEF) treatment is an effective process for preservation of liquid products in food and biotechnology at reduced temperatures, by causing electroporation. It may contribute to increase retention of heat-labile constituents with similar or enhanced levels of microbial inactivation, compared to thermal processes. However, especially continuous PEF treatments suffer from inhomogeneous treatment conditions. Typically, electric field intensities are highest at the inner wall of the chamber, where the flow velocity of the treated product is lowest. Therefore, inhomogeneities of the electric field within the treatment chamber and associated inhomogeneous temperature fields emerge. For this reason, a specific treatment chamber was designed to obtain more homogeneous flow properties inside the treatment chamber and to reduce local temperature peaks, therefore increasing treatment homogeneity. This was accomplished by a divided inlet into the chamber, consequently generating a swirling flow (vortex). The influence of inlet angles on treatment homogeneity was studied (final values: radial angle α = 61°; axial angle β = 98°), using computational fluid dynamics (CFD). For the final design, the vorticity, i.e., the intensity of the fluid rotation, was the lowest of the investigated values in the first treatment zone (1002.55 1/s), but could be maintained for the longest distance, therefore providing an increased mixing and most homogeneous treatment conditions. The new design was experimentally compared to a conventional co-linear setup, taking into account inactivation efficacy of *Microbacterium lacticum* as well as retention of heat-sensitive alkaline phosphatase (ALP). Results showed an increase in *M. lacticum* inactivation (maximum Δlog of 1.8 at pH 7 and 1.1 at pH 4) by the vortex configuration and more homogeneous treatment conditions, as visible by the simulated temperature fields. Therefore, the new setup can contribute to optimize PEF treatment conditions and to further extend PEF applications to currently challenging products.

## Introduction

Pulsed electric fields (PEF) treatment is a process increasingly used in food and biotechnology, as the resulting electroporation can cause damage to membranes of biological cells, which consequently enables a variety of different applications including gene transfer, enhancement of mass transfer and extraction, as well as non-thermal decontamination with a reduced thermal load (Kotnik et al., 2015).

For the continuous PEF treatment of liquids, the most commonly used type of treatment chamber is the so-called co-linear electrode configuration, involving ring-shaped electrodes and insulators in an alternating order. This configuration is characterized by a relatively high electrical resistance, thus enabling high electric field strength levels necessary for many cell disruption tasks while limiting electrical current flow, energy input and the associated temperature increase ($\Delta \,T=W_{s\,p\,e\,c}\,c_{p}^{-1}$; with Δ*T*: temperature increase, *W*_*spec*_: specific electric energy input, *c*_*p*_: specific heat capacity at constant pressure), especially for products with a higher electrical conductivity (Toepfl, 2006). However, due to this design, the electric field is not equally distributed within the chamber. In fact, highest current densities are present at the inner walls of the chamber (Jaeger et al., 2009; Reineke et al., 2015; Woelken et al., 2017). Moreover, as treatments usually operate under laminar flow conditions, this also corresponds to the position with the lowest flow velocity, therefore leading to local electric field and temperature peaks, accompanied by possible negative effects on heat-sensitive compounds of the product to be treated. This is of special relevance for the reduction of the microbial load in bioactive products, like protein or enzyme solutions (Schottroff et al., 2019), as the occurring electric field and temperature inhomogeneities can contribute to a reduced inactivation of microorganisms, and an increased thermal destruction of valuable compounds, respectively. Especially at neutral pH, without the presence of additional anti-microbial hurdles (Leistner, 1995) and for challenging products with higher viscosities or contents of protective ingredients, this can have pronounced negative implications on the quality of the treated product, as a high level of energy input and an associated occurrence of pronounced hot spots may be necessary to achieve the desired microbial log reduction. Limitations regarding the processability of such products and negative implications on bioactive compounds have been shown earlier (Schottroff et al., 2020).

Some approaches have already been undertaken to improve homogeneity during continuous PEF treatment or to reduce thermal effects, e.g., by optimization of insulator designs (Buckow et al., 2011), use of a static mixer (Jaeger et al., 2009), or electrode cooling (Saldaña et al., 2010; Meneses et al., 2011a). However, none of these approaches have been implemented on an industrial scale so far, due to mechanical stress and reduced cleaning-in-place ability of static mixers and limitations in upscaling considering heat transfer of cooled electrodes.

In order to overcome these limitations, a so-called vortex treatment chamber was designed and optimized using CFD, aiming to improve mixing inside the chamber, but also being scalable. For this purpose, the flow-through chamber was planned in such a way that the product inlet was divided and shifted, so that the product was inserted into the chamber at a certain angle, creating a swirling flow which should affect the exposure of individual fluid volume elements to the electric field. As a result, the temperature peaks within the treatment chamber were supposed to be reduced. This was verified by numerical simulations of the flow, electric field and temperature distributions. Based on these outcomes, inactivation of the heat-resistant bacterium *Microbacterium lacticum* and the heat-sensitive enzyme alkaline phosphatase (ALP) was investigated. Microbial inactivation levels and retention of bioactivity of the enzyme were subsequently evaluated. Thus, the vortex design was experimentally characterized, in comparison to the current standard-configuration, i.e., the co-linear treatment chamber, in order to proof the concept of more homogeneous treatment conditions in a continuous swirling flow PEF chamber.

## Materials and Methods

### Standard and Modified Chamber Geometry

**FIGURE 1:**
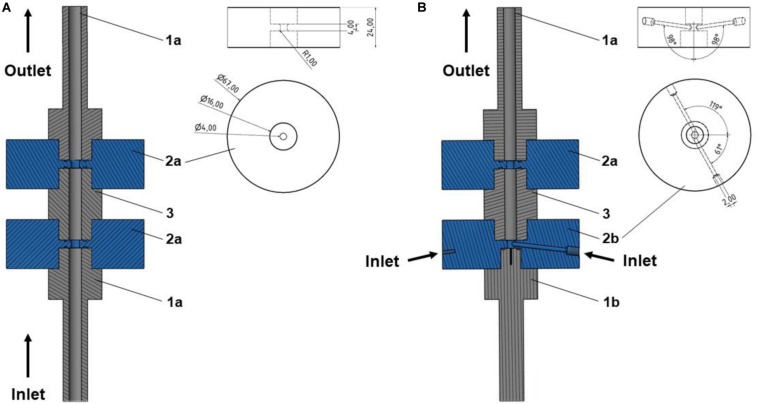
Cross sections of the two different designs used in this study, i.e., co-linear **(A)** and vortex **(B)** PEF treatment chamber configurations, including the final dimensions. Depicted are the flow-through ground (1a) and high voltage (3) electrodes, and disk-shaped flow-through insulators (2a). For the vortex configuration, a solid ground electrode (1b) and the specific bottom insulator design (2b) were implemented. The remaining configuration was unchanged. More information on the co-linear chamber and further dimensions are provided by Meneses et al. (2011a).

Two different PEF treatment chamber configurations were used in this study – the standard co-linear treatment chamber as well as the newly developed vortex chamber. Both chambers consist of an alternating sequence of electrodes and insulators. The center of each treatment chamber is formed by the hollow high voltage electrode, which is surrounded by two hollow disk-shaped insulators and two terminal ground electrodes, at the inlet or outlet, respectively. The two treatment chambers differ by the design of the inlet, i.e., the bottom ground electrode as well as the first insulator, with the remaining configuration being identical (Figure 1).

**FIGURE 2:**
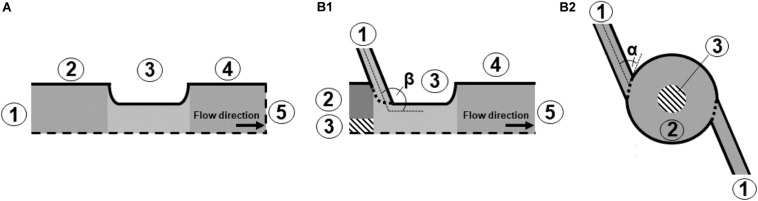
Schematic drawing of the simulated domain for co-linear **(A)** and vortex **(B1)** configurations, and bottom view of the vortex treatment chamber **(B2)**. Further depicted are the varied inlet angles, α and β, as well as the boundaries (1–5). The values of the boundaries are summarized in Table 2. The dashed lines indicate symmetry lines.

Figure 2 depicts a schematic of the standard co-linear treatment chamber and a base configuration of the vortex treatment chamber. The co-linear treatment chamber has an inner pipe diameter of 6 mm that is reduced to 4 mm within the insulators. The chamber consists of two identical treatment zones, i.e., the volume inside the insulators where the electric field exposure takes place, with a length of 4 mm each, see also Figure 1.

The modified chamber geometry differs therefore in the design of the first treatment zone. In order to enhance the homogeneity of the treatment, the central inflow was split into two inlets through which the liquid was introduced into the bottom insulator. This configuration causes a rotation of the liquid within the chamber and potentially reduces over-processing in stagnation zones. Each inlet is characterized by two angles, α and β, which determine the position of the respective inlet pipes (2 mm inner diameter each) with respect to the chamber. The second treatment zone is identical to the co-linear treatment chamber (Figure 1). For both chambers, the total treatment volume inside the two insulators was 100.5 μL.

### Thermofluiddynamical Model of the PEF Process

In order to simulate the PEF process, the bacterial and enzyme suspensions were treated as incompressible, single-phase fluids, i.e., the effect of the cells and molecules on the flow, temperature, and electric field was neglected. This assumption is justified due to the low volume fraction of cells and enzymes, their small size and consequently their negligible impact on the transport of momentum and energy (Crowe et al., 2011). Moreover, for the mass flows under consideration, laminar flow conditions can be expected for all investigated conditions. This estimate is based on the magnitude of the Reynolds number within the smallest and largest diameters of the geometry and the consideration of the respective mean flow velocities. Based on these estimates, the thermofluiddynamical model for the PEF treatment contains equations for the conservation of mass, momentum, energy, and electric charges. In addition, a first order kinetic model for thermal inactivation was included in order to estimate the contribution of thermal effects on the overall inactivation during the treatment. The equation of mass conservation for an incompressible fluid reads

(1) ∇⋅u=0,

where **u** is the fluid velocity vector. The equation for momentum conservation reads

(2) $$\frac{\partial \rho \,\text{u}}{\partial t}+\nabla \cdot \left(\rho \,{\text{uu}}^{T}\right)=-\nabla p+\nabla \cdot \tau +\rho \,\text{g}.$$

Herein, *t* stands for time, ρ for mass density of the fluid, *p* is the local static pressure, **g** the vector of gravitational acceleration and τ the stress tensor, which for an incompressible fluid is given as

(3) $$\tau =\mu \,\left(\nabla \text{u}+{\left(\nabla \text{u}\right)}^{T}\right),$$

where μ is the dynamic viscosity. The energy conservation equation reads

(4) $$c_{p}\,\frac{\partial \rho \,T}{\partial t}+c_{p}\,\nabla \cdot \left(\rho \,\text{u}\,T\right)=\nabla \cdot \left(\lambda \,\nabla T\right)+\tau_{p}\,f_{p}\,\sigma \,E^{2}.$$

Herein, *T* is the local absolute temperature, *c*_*p*_ the specific heat capacity of the fluid and λ the thermal conductivity. The last term of Eq. 4 covers the Joule heating of the liquid during the electric pulse. The rate of energy production is given as the product of pulse width τ_*p*_ and pulse repetition rate *f*_*p*_ of the electric pulse, local electrical conductivity σ and the squared local electric field strength *E*. The latter can be calculated by solving the conservation equation of electric charges, which reads

(5) ∇⋅(σ⁢∇⁡Φ)=0.

In Eq. 5, Φ is the electric potential, which is related to the electric field strength by

(6) E=-∇⁡Φ.

Additionally, the thermal inactivation of the used microbial cells and enzymes was modeled. Therefore, a transport equation for the residual activity *RA* of the biological species was considered (Rauh et al., 2009), which reads

(7) $$\frac{\partial R\,A}{\partial t}+\nabla \cdot \left(\text{u}\,R\,A\right)=r_{R\,A}$$

where *r*_*RA*_ is a reaction term considering the reduction of *RA* by thermal inactivation. The reaction is modeled as a first order kinetic, as described elsewhere (Jaeger et al., 2010; Dumitraşcu et al., 2015), thus

(8) $$r_{R\,A}=-k\,\left(T\right)\,R\,A.$$

The kinetic parameter *k*(*T*) includes the temperature dependency of the inactivation rate by the Arrhenius equation:

(9) $$k\,\left(T\right)=\frac{2.303}{D_{T_{r\,e\,f}}}\,\exp\left(-\frac{2.303\,T^{2}}{z}\,\left(\frac{1}{T}-\frac{1}{T_{r\,e\,f}}\right)\right)=k_{0}\,\exp\left(-\frac{E_{\alpha }}{R\,T}\right)$$

Here, *D*_*T_ref*_ represents the D-value at reference temperature *T*_*ref*_ and *z* is the z-value of *M. lacticum* (see section “Microbiological Procedures”) or ALP (see section “Quality Analysis”), respectively. The definitions of D and z-values are given in Eqs. 10 and 11. With this regard, the D-value represents the decimal reduction time, i.e., the time needed at a constant temperature *T* to reduce the initial microbial population (*N*_0_) by 90%, taking into account treatment time *t* and the corresponding inactivation (*N*_*t*_). In a similar manner, the z-value describes the temperature increase or decrease needed to reduce or raise the inactivation time, i.e., the D-value, by 90%, based on a reference temperature *T*_*ref*_ and another temperature level *T*, as well as the corresponding D-values, *D*_*T_ref*_ and *D*_*T*_. Moreover, the activation energy *E*_*a*_ (see Eq. 9) was derived from the Arrhenius plot, from the natural logarithm (ln *k*(*T*)) of the individual, temperature-dependent rate constants (Eq. 12) plotted over 1/*T*. In this context, *k*_*o*_ refers to the rate constant for 1/*T*→0 and *R* gives the universal gas constant, i.e., 8.314 J/(mol K).

(10) $$D_{T}=\frac{t}{\log N_{0}-\log N_{t}}$$

(11) $$z=\frac{T-T_{r\,e\,f}}{\log D_{T_{r\,e\,f}}-\log D_{T}}$$

(12) $$k_{T}=\frac{2.303}{D_{T}}$$

The material properties of the liquid used for the simulations are summarized in Table 1. In this regard, all material properties except from the electrical conductivity were assumed to be constant and independent from temperature. In case of the electrical conductivity, a linear relationship with respect to the temperature was assumed (Woelken et al., 2017), according to experimental measurements. Consequently, the electric and temperature fields were coupled to each other.

**TABLE 1:** Material properties of the Ringer’s solution used as the treatment medium for the trials, as deployed in the CFD simulation.

Property	Symbol	Value	Unit
Density	ρ	999.9	*kg*/m^3^
Viscosity	μ	1.0	mPas
Heat capacity	*c*_*p*_	4184.52	J/(*kgK*)
Thermal conductivity	λ	599.35×10^–3^	W/(*mK*)
Electrical conductivity	σ(*T*)	0.0073*T*[°*C*] + 0.1874	S/m

*Values derived from VDI Heat Atlas (VDI, 2010), conductivity was self-determined.*

**TABLE 2:** Boundary conditions on the positions indicated by the numbers in Figure 2.

Boundary and number	Condition
**Flow model**	
Inlet (1)	$u_{i\,n}=\frac{4\,\bar{V}}{\pi \,d_{i\,n\,l\,e\,t}^{2}}$
Outlet (5)	*p*_*stat*_−*p*_*ref*_ = 0
Walls (2, 3, 4)	**u** = 0
**Thermal model**	
Inlet (1)	*T* = *T*_0_
Outlet (5)	∇⁡*T* = 0
Walls (2, 3, 4)	*q* = −λ∇⁡*T* = 0
**Electrostatic model**	
High voltage electrode (4)	*U* = *U*_0_
Grounding (2)	*U=0*
Insulator (3)	σ∇⁡ϕ = 0
Inlet (1)	σ∇⁡Φ = 0
Outlet (5)	σ∇⁡Φ = 0
**Inactivation model**	
Inlet (1)	*RA* = 1
Outlet (5)	∇⁡*RA* = 0
Walls (2, 3, 4)	*q*_*RA*_ = 0

Eqs. 1–7 can be solved numerically with proper boundary conditions, which are summarized in Table 2. The positions of the boundaries are shown by their numbers in Figure 2. In brief, the fluid is assumed to flow into the treatment chamber with a parabolic velocity profile, corresponding to the average velocity *u*_*in*_, and a constant temperature. The walls of the chamber are considered adiabatic, which is a reasonable assumption, since they are usually covered by an insulating material. In addition, the common no-slip condition is applied at the walls. A constant pressure and a vanishing temperature gradient are assumed at the outlet of the chamber. For the electric field, the voltage at the high voltage and grounding electrodes were set to *U* = *U*_0_ and *U* = 0 V, respectively. At the chamber inlets and outlets, as well as at the insulating walls of the chamber, a zero-gradient condition was set for the electric potential. For the residual activity, a value of *RA* = 1 was set at the chamber inlet, which is equivalent to *log*⁡(*N*/*N*_0_). At the outlet, a zero gradient condition was set for the residual activity.

In order to solve Eqs. 1–7 numerically, the domain was discretized after a grid convergence study in 810,812 volume elements by means of the software Ansys Meshing 19.2. The solution of the model was obtained by transient simulations of the flow with the finite volume code ANSYS CFX 19.2 (ANSYS, Inc., Canonsburg, United States). The simulations where initialized with a steady-state solution of the flow and subsequently improved by performing a time-resolved transient simulation with similar conditions. The total simulation time was set to 3 s, which is approximately four times the average residence time of a fluid element in the treatment chamber. After this time, the properties of the flow changed less than 0.5% within a time period of 0.2 s, so that the flow and the treatment conditions can be assumed as steady and therefore representative for the treatment.

### Optimization of Chamber Design

Simulation studies were performed in order to determine the design of the new treatment chamber. This was done by a systematic variation of the angle α between 61° and 90°, while β was held constant at 98° because of manufacturing restrictions. This is supported by preliminary simulation studies in which no clear relation of the treatment conditions and β was detected.

For the assessment of the different geometries, three major quantities were evaluated. According to the working hypothesis, a large magnitude of the vorticity

(13) ω=|∇×u|

corresponds to a more homogeneous treatment. As an integral measure for the vorticity, the volumetric average was calculated within each of the two treatment zones of the chamber geometry, which are indicated by the light gray area in Figure 1. From an integral point of view, the total specific energy input

(14) $$W_{s\,p\,e\,c}=\frac{1}{\dot{m}}\,f_{p}\,\tau_{p}\,\int_{V}\sigma \,E^{2}\,dV$$

and the pressure loss between the inlet and outlet of the chamber

(15) $$\Delta \,p=\left(p_{i\,n}-p_{o\,u\,t}\right)+\frac{\rho }{2}\,\left(u_{i\,n}^{2}-u_{o\,u\,t}^{2}\right)$$

are important quantities and therefore evaluated for the different treatment chambers. It should be noted that *W*_*spec*_ only differs between the different designs because of the temperature-dependency of the electrical conductivity.

### Prototyping

Based on the obtained results of the simulation, a prototype of the vortex treatment chamber configuration was designed using 3D CAD (Inventor, Autodesk, Corp., San Rafael, United States). For reasons of comparability, the co-linear configuration was modified accordingly (see Figure 1). For this purpose, the hollow flow-through ground electrode was replaced by a solid bottom electrode of the same dimensions, including a non-conductive center (Acrifix, Evonik Performance Materials GmbH, Darmstadt, Germany) of 2 mm diameter, to limit the present currents, as determined by simulation. The inlet of the cell was implemented through the bottom insulator, by two opposing inlet holes of 2 mm diameter each, compared to 4 mm inner diameter of the insulator. The inlet drillings featured the determined angles (α and β). The corresponding inlet tubing (Festo AG & Co. KG, Esslingen, Germany) was connected via screw-in hose nozzles (Festo AG & Co. KG, Esslingen, Germany). The further parts and dimensions of the treatment chamber were identical to those of the co-linear configuration (see Meneses et al., 2011a for further details and dimensions). All electrodes were produced from V2A stainless steel, the insulators consisted of polyoxymethylene (POM).

### Microbiological Procedures

*Microbacterium lacticum* D84 (EF204392), an isolate from heat-treated extended shelf life milk, was used as a heat-resistant model bacterium for the microbiological trials. This strain was determined to be the most heat resistant from a set of available vegetative microorganisms in preliminary screening studies, including six different heat-resistant *M. lacticum* isolates, based on determination of D-values (data not shown). Due to the pronounced heat resistance, *M. lacticum* is a suitable indicator for electroporation phenomena within a wide temperature range, since superimposing thermal inactivation effects become relevant at higher temperatures only.

A single colony of *M. lacticum* was inoculated in 1 mL of tryptic soy broth (TSB; VWR International SPRL/BVBA, Leuven, Belgium) and incubated over night at 37°C. Subsequently, this overnight culture was inoculated 1:100 in TSB and incubated for 24 h at 37°C without shaking, to obtain cells in the early stationary phase. This working culture was centrifuged at 2700 *g* for 10 min at 20°C and washed three times in $\frac{1}{4}$ strength Ringer’s solution (Merck KGaA, Darmstadt, Germany). Final inoculation was carried out in appropriate amounts of sterile, diluted Ringer’s solution at pH 4 and 7, with a defined electrical conductivity of 3.0 mS/cm. The pH was adjusted using 10% lactic acid (Sigma Aldrich, St. Louis, MO, United States). Initial counts were in the range of 10^7^ colony forming units (CFU) per mL.

After each treatment, 50 μL of the untreated (*N*_0_) as well as the treated (*N*) microbial suspensions were plated on tryptic soy agar (TSA) in duplicate. Plates were incubated at 37°C for 48 h. Cell counts were consecutively determined and inactivation levels were expressed as log_10_(*N*/*N*_0_). In the following parts of the manuscript, log_10_ will simply be referred to as log. All trials were carried out in triplicate.

### Quality Analysis

In order to determine the effects of temperature during continuous PEF treatment, bovine alkaline phosphatase (ALP; CAS number 9001-78-9, AppliChem GmbH, Darmstadt, Germany), an enzyme found, e.g., in milk, was exemplarily used as a representative for heat-sensitive bioactive molecules. ALP was dissolved in diluted Ringer’s solution (3.0 mS/cm) at pH 4 and 7. Enzyme activity was determined using a colorimetric assay (Amplite Alkaline Phosphatase Assay Kit, AAT Bioquest, Sunnyvale, CA, United States) according to the manufacturer’s specifications. Enzyme activity was expressed as mU/mL and enzyme inactivation was calculated as the residual activity (*RA*), i.e., as *A*/*A*_0_, from the initial (*A*_0_) activity and the activity after the treatment (*A*). Initial enzyme activity was 160 mU/mL. All trials and analyses were carried out in triplicate.

### Thermal Reference Data

In order to obtain data on the heat sensitivity of *M. lacticum* and ALP, thermal reference trials were carried out, using different temperature-time combinations. This was accomplished by enclosing 80 μL of microbial suspension or enzyme solution in small glass capillaries (*d*_*i*_ = 1 mm, *d*_*a*_ = 1.3 mm, *L* = 100 mm, Kleinfeld Labortechnik GmbH, Gehrden, Germany). Capillaries were heat-sealed using a bunsen burner, while simultaneously being cooled (Haas et al., 1996; Reineke et al., 2015). Capillaries were immersed into a water bath at the respective temperature for predefined holding times and immediately cooled in ice water after the treatment. Capillaries were opened by means of disinfected pliers, the contents were withdrawn using a 200 μL pipette and analyzed as mentioned above.

Thermal-only inactivation data were linearly modeled and mathematically described, using D and z-values (Eqs. 10 and 11) for microorganisms, values for ALP were calculated analogously, by replacing the microbial counts with the corresponding *RA*.

### Characterization of the Newly Developed and Standard Co-linear Treatment Chambers

For the evaluation of the two treatment chamber configurations, the vortex and co-linear designs were experimentally compared, considering their similarity in residence time behavior as well as their individual efficacy for inactivation and retention of heat-sensitive matrix compounds.

#### Continuous Treatments

**FIGURE 3:**
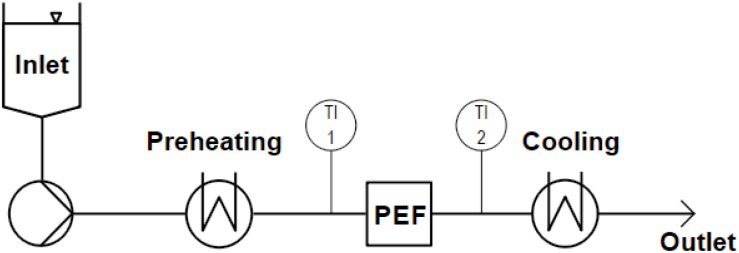
Scheme of the used continuous PEF equipment, including an inlet tank, a screw pump, heat exchangers for preheating and cooling, inlet (TI 1) and outlet (TI 2) temperature sensors, and the PEF treatment chamber (co-linear or vortex configuration).

The used continuous PEF setup (Figure 3) consisted of a screw pump with an inlet vessel (MDC 006-12, Seepex GmbH, Bottrop, Germany), coil heat exchangers for preheating and cooling (self-built) and the co-linear or vortex PEF chamber. Inlet and outlet temperatures were measured before and 110 mm after the treatment chamber, in the center of the pipe. The system is described in more detail by Schottroff et al. (2019).

As the electric field inside of flow-through treatment chambers is inhomogeneously distributed (Toepfl, 2006; Jaeger et al., 2009), a trivial relation of voltage and electrode distance is inadequate to describe the present electric field strength (*E*) during the treatment. Therefore, an average electric field strength (*E*_*avg*_) can be derived from simulations, employing a design-specific conversion factor (*c*_*chamber*_), which enables the calculation of *E*_*avg*_ from the applied voltage (Eq. 16). For the used co-linear treatment chamber configuration, *c*_*chamber*_ amounts to 1.6 (Jaeger et al., 2009). The setup was designed in such a way that the average electric field strength remained constant for both treatment chamber configurations (Table 3).

**TABLE 3:** Used PEF parameters for the different trials, including voltage (*U*), average electric field strength (*E*_*avg*_), maximum current (*I*_*max*_), pulse width (τ_*p*_), maximum pulse repetition rate (*f*_*p*,*max*_), used mass flow ($\dot{m}$), inlet temperature (*T*_*in*_) as well as maximum outlet temperature (*T*_*out*,*max*_).

PEF chamber	*U*_*0*_ [kV]	*E*_*avg*_ [kV/cm]	*c*_*chamber*_ [1/cm]	*I*_*max*_ [A]	τ_*p*_ [μs]	*f*_*p*,*max*_ [Hz]	$\dot{m}$ [kg/h]	*T*_*in*_ [°C]	*T*_*out*,*max*_ [°C]
Co-linear	20	32	1.60	62	3	130	7.0	50	87
Vortex	18.1	32	1.77	62	3	130	7.0	50	87

*Further given are the conversion factors (*c*_*chamber*_) for the determination of average electric field strength from the applied voltage, depending on the individual treatment chamber design. Value for the co-linear chamber was determined by Jaeger et al. (2009).*

(16) $$E_{a\,v\,g}=c_{c\,h\,a\,m\,b\,e\,r}\,U$$

For the trials, the continuous system was started using saline solution of the same electrical conductivity than the *M. lacticum* suspensions or ALP solutions (3.0 mS/cm), and once a steady state was reached, the product inlet was changed to the liquid to be treated. After the determined residence time (see section “Overall Residence Time Distribution”), samples were gathered and placed on ice until further analysis. In-between parameter changes, a corresponding fraction of the residence time had to pass by. Details on the setup of the process are reported elsewhere (Schottroff et al., 2019). The used process parameters are given in Table 3.

#### Overall Residence Time Distribution

Residence time distribution profiles were obtained in both setups. After connection of the individual treatment chamber, the pump was started with tap water and the inlet was abruptly changed to saline solution of a defined electrical conductivity (3.0 mS/cm). From this starting point on, samples were taken every 30 s and the electrical conductivity was determined (EL3 handheld conductivity meter, Mettler Toledo, Columbus, United States). By interpretation of the electrical conductivity as the concentration of ions, *c*(*t*), the dimensionless cumulated residence time *F*(*t*) as well as the residence time distribution function *E*_*r*_(*t*) could be calculated (Eqs. 17 and 18). From these curves, the mean residence time $\bar{t}$ (Eq. 19), as well as the variance $\sigma_{v\,a\,r}^{2}$ were derived (Eq. 20). In Eqs. 17–20, *c* stands for concentration and *t* gives the time. Subscripted 0 refers to initial values.

(17) $$F\,\left(t\right)=\frac{c\,\left(t\right)}{c_{0}}$$

(18) $$E_{r}\,\left(t\right)=\frac{d\,F\,\left(t\right)}{d\,t}$$

(19) $$\bar{t}=\int_{0}^{\infty }t\,E_{r}\,\left(t\right)\,dt$$

(20) $$\sigma_{v\,a\,r}^{2}=\int_{0}^{\infty }t^{2}\,E_{r}\,\left(t\right)\,dt-{\bar{t}}^{2}$$

### Differentiation of Thermal and Electric Field Effects

In order to evaluate the individual thermal and electric field effects of the two treatment chamber configurations, the thermal-only inactivation of *M. lacticum* or ALP during the treatment was calculated by solving the thermofluiddynamical model as given by Eqs. 1–9, under consideration of the respective measured thermal reference data. The effects of the electric field alone could consequently be evaluated from the difference of experimentally determined inactivation (which is a mixture of thermal and electric field effects) and the calculated thermal-only inactivation, see Eq. 21 (Jaeger et al., 2010; Reineke et al., 2015). Inactivation of ALP was calculated equivalently.

(21) $$\log{\left(\frac{N}{N_{0}}\right)}_{P\,E\,F}=\log{\left(\frac{N}{N_{0}}\right)}_{e\,x\,p\,e\,r\,i\,m\,e\,n\,t\,a\,l}-\log{\left(\frac{N}{N_{0}}\right)}_{t\,h\,e\,r\,m\,a\,l}$$

### Data Processing, Visualization, and Statistical Analysis

Analytical data were processed in Microsoft Excel (Microsoft, Corp., Redmond, WA, United States) and visualized using SigmaPlot 14 (Systat Software, Inc., San Jose, CA, United States). Statistical analyses (Student’s *t*-test) were performed using Statgraphics Centurion XVII (Statpoint Technologies, Inc., Warrenton, VA, United States).

## Results and Discussion

### Thermal References

*Microbacterium lacticum* and ALP were thermally inactivated in order to obtain data for the simulation of thermal-only effects during PEF treatment. The correspondingly determined D and z-values, as well as activation energies are displayed in Table 4. Considering the values for *M. lacticum*, inactivation took 122% longer at neutral pH than at pH 4 (180.6 vs. 81.2 s) at 80°C. This effect was distinctly less pronounced for higher temperatures, where slightly increased values were observed, i.e., 48% at 85°C (28.8 vs. 19.4 s) and 16% at 90°C (3.6 vs. 3.1 s). The z-values, on the other hand, were relatively similar, with values of 7.0 and 5.9°C at pH 4 and 7, respectively. Likewise, the activation energy at pH 4 (350.1 kJ/mol) was lower than at pH 7 (393.8 kJ/mol), thus also indicating a facilitated inactivation at pH 4. The reason for this increased inactivation is most likely a consequence of the additional antimicrobial hurdle present in form of the low pH in combination with heat (Leistner, 1995). On the other hand, it was observed that a longer contact time (approximately > 1 h) of bacteria and low-pH medium led to a drastic increase of the heat resistance, implying the occurrence of cross-protective effects (Ryu and Beuchat, 1998). Therefore, the contact time was carefully considered for the trials and kept below 30 min.

**TABLE 4:** Thermal inactivation data of *M. lacticum* and ALP.

Indicator	pH [−]	*T* [°C]	*D*_*T*_ [s]	*z* [°C]	*E*_*a*_ [kJ/mol]
*M. lacticum*	4	80	81.2	7.0	350.1
		85	19.4		
		90	3.1		
	7	80	180.6	5.9	393.8
		85	28.8		
		90	3.6		
ALP	4	67	312.5	13.0	180.1
		72	277.8		
		77	28.6		
		82	36.6		
		87	10.4		
	7	67	256.4	13.9	169.3
		72	185.2		
		77	37.7		
		82	29.9		
		87	10.0		

*Values were determined as described in section “Thermal Reference Data.”*

Considering ALP inactivation, it was observed that enzyme activity was lower at pH 4, due to the severe deviation from the optimum pH range of 7–9 (Ross et al., 1951). However, by expressing enzyme inactivation based on the initial activity, this fact could be compensated for the calculation of the kinetics. In contrast to *M. lacticum*, thermal inactivation of ALP was slightly increased at neutral pH, compared to pH 4, although the differences between the two values were not as pronounced as for the bacterium. Increase of inactivation was in the range of 33% (72°C; 277.8 vs. 185.2 s) to 3.5% (87°C; 10.4 vs. 10.0 s), based on the comparison of D-values. The z-values also only differed by 5.9%, with values of 13.0°C and 13.9°C, for pH 4 and 7, respectively. Moreover, the activation energy of 169.3 kJ/mol at pH 7 also indicates a facilitated enzyme inactivation at that pH level, compared to pH 4 (180.1 kJ/mol; Δ*E*_*a*_ of 6.4%).

These values were further used for the calculation of thermal inactivation occurring in the individual PEF chambers (see section “Thermofluiddynamical Model of the PEF Process”).

### Design Optimization of the Vortex Treatment Chamber

**FIGURE 4:**
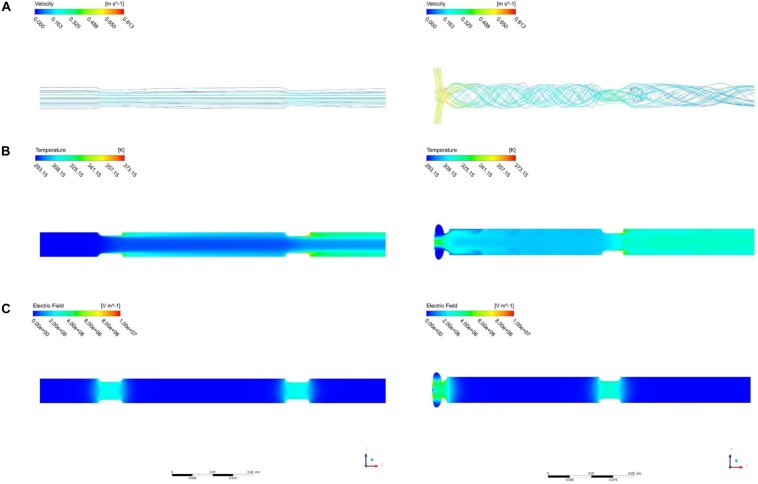
Comparison of the co-linear (left) and the vortex (right, α = 61°) treatment chambers, including velocity streamlines **(A)** with colors indicating the fluid velocity, temperature distribution **(B)**, as well as electric field strength **(C)**. Assumed processing conditions: $\dot{m}=10.2\,\mathrm{kg}/h$, *T*_*in*_ = 20°C, *E*_*avg*_ = 32*kV*/*cm*, *f*_*p*_ = 105Hz, τ_*p*_ = 3μs. Note that the scale on the left and right side of the figure is different, as indicated by the scale bar at the bottom.

Figure 4 compares the simulated features of PEF processing between the vortex chamber design and the common co-linear design. The assumed processing conditions for the simulations were a liquid mass flow $\dot{m}=10.2\,\mathrm{kg}/h$, liquid temperature *T*_*in*_ = 20°C, applied voltage *U*_0_ = 20kV, pulse repetition rate *f*_*p*_ = 105Hz, and pulse width τ_*p*_ = 3μs. The set of parameters represents typical conditions for a pilot-scale PEF treatment and was therefore chosen as a test case for the comparison of the treatment chamber characteristics. The computed flow and temperature patterns in the co-linear chamber agree to the descriptions in the literature (Jaeger et al., 2009; Woelken et al., 2017) and are characterized by parallel streamlines and the formation of a liquid jet behind the treatment zones. This flow pattern creates a recirculation zone behind the insulators, where local temperature maxima can be found. Besides, the temperature is the lowest in the center of the chamber because of the faster convective transport of thermal energy in the direction of the liquid flow. In the modified treatment chamber, a swirling flow regime was created, as expected. The swirling flow was even maintained within and behind the second treatment zone so that no recirculation zones evolved, unlike the co-linear chamber. Consequently, the temperature distribution was distinctly more homogeneous across the cross-section of the pipe in the whole treatment chamber. The distribution of the electric field in the second treatment zone is similar in both chambers due to the equivalent design. In contrast, the electric field strength in the first treatment zone is found to be higher in the vortex design, due to the different arrangement of the electrodes.

Table 5 summarizes the conditions in the treatment chamber for different values of the angle α. For comparison, simulation results for the conventional co-linear configuration are also reported. The swirling flow can be characterized in terms of the vorticity, which is a measure of the strength of the fluid rotation. According to the simulated results, the average vorticity in the first treatment zone becomes the highest for α = 65° (1320.12 1/s). Among the different simulated designs, the vorticity in the first treatment zone varied by 25% with respect to the maximum value, whereas the minimum value was found for α = 61° (1002.55 1/s). However, it can also be observed that for α = 61° the swirling flow can be maintained for a longer period, which is reflected by the highest vorticity in the second treatment zone (434.16 1/s), while it is the lowest for α = 65° (260.26 1/s). Generally, it was observed that the difference of the average vorticity between treatment zones 1 and 2 is linearly correlated with the average vorticity in the first treatment zone (*R*^2^ = 0.9946), or, in other words, the higher the average vorticity in the first treatment zone the faster it decays within the treatment chamber. This also corresponds to the estimated pressure loss over the treatment chamber, which is positively correlated to the average vorticity in the first treatment zone. Therefore, under the simulated conditions, the maximum pressure loss was found for **α = 65°** (367.05 Pa), while the pressure loss for α = 61° was about 20% lower (296.31 Pa). The pressure loss of the co-linear treatment chamber was estimated to be in a similar order of magnitude (327.54 Pa).

**TABLE 5:** Characteristics of the flow and treatment conditions in the two different treatment chambers.

Design	Angle α [°]	*W*_*spec*_ [kJ/kg]	Δ*T* [K]	ω [1/s] Zone 1	ω [1/s] Zone 2	Δω [1/s]	Δ*p* [Pa]
Vortex	90	103.51	24.06	1170.41	331.03	389.38	324.14
	85	103.27	24.23	1184.92	319.47	865.45	326.01
	80	102.87	24.41	1211.37	302.01	909.36	331.12
	75	102.79	24.05	1222.30	287.41	934.87	341.66
	70	102.56	24.47	1249.92	266.76	983.16	353.86
	65	101.00	24.73	1320.12	260.21	1059.91	367.05
	61	105.03	24.84	1002.55	434.16	568.39	396.31
Co-linear	–	76.64	21.75	177.16	206.03	−28.87	327.54

In comparison to the co-linear chamber (76.64 kJ/kg), a significantly higher specific energy input *W*_*spec*_ was observed in all configurations of the modified chamber (103.37–105.12 kJ/kg) if the applied voltage is similar in both chambers. This observation can be explained by the more homogeneous temperature distribution in the modified treatment chamber, as it increases the electrical conductivity in the treatment zone and therefore the current and the associated energy input in areas of high electric field strength. The results show that the general design of the chamber is more important than the impact of the angle α on the overall energy input, as *W*_*spec*_ differs less than 1.6% between all configurations of the vortex treatment chamber but about 25% between the conventional co-linear and the vortex designs.

The simulation results indicate that the vortex treatment chamber is superior over the co-linear treatment chamber. In accordance to the obtained results, the chamber geometry with α = 61° was chosen for the experimental investigations, since it unifies the highest specific energy input and the lowest pressure loss, which are both important parameters for future industrial applications.

Previously published studies on improvement of treatment homogeneity during PEF processing optimized the inner insulator geometry (Buckow et al., 2011; Meneses et al., 2011b; Woelken et al., 2017), applied electrode cooling (Meneses et al., 2011a), or implemented a static mixer in the treatment chamber (Jaeger et al., 2009). The treatment chambers investigated were already equipped with an optimized insulator geometry, as described by Meneses et al. (2011b). Although the electric field and flow are influenced to a certain extent, there was still room for improvement. Electrode cooling was not further considered, as especially for upscaling applications the heat transfer was determined to be insufficient (data not shown). Moreover, a static mixer was investigated in preliminary studies, but also turned out to be impracticable due to reduced cleanability (data not shown). Therefore, the vortex treatment chamber configuration is a promising approach for improvement of treatment homogeneity, while compensating the above-mentioned issues.

### Characterization and Comparison of the Used Setups

**FIGURE 5:**
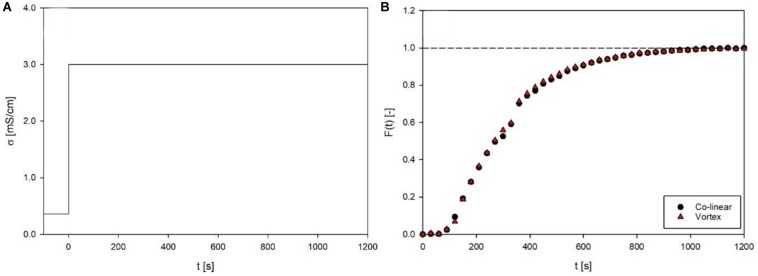
Inlet function **(A)** and response function **(B)** of the residence time distribution for co-linear and vortex chambers at a flow rate of 7 L/h. Given are electrical conductivity (σ) and the calculated dimensionless residence time function *F*(*t*) over time. Data points depict the average of three independent replicates.

The overall residence time distribution between inlet and outlet of the continuous PEF system (Figure 3) was evaluated, in order to ensure comparable treatment conditions for the two different process setups. Both setups showed a distinctly similar, almost identical residence time behavior (Figure 5), with mean residence times $\bar{t}$ of 316.4 s and 312.2 s for the co-linear and vortex-chamber, respectively, and corresponding variance $\sigma_{v\,a\,r}^{2}$ of 37321.4 s^2^ and 35945.8 s^2^. The overall mean residence time of the vortex configuration was therefore 4.2 s shorter compared to the co-linear configuration. This could be due to the accomplished alteration of the inlet, using small plastic tubes (see section “Prototyping”), compared to the little larger metal tubing of the co-linear configuration (see Figure 1). However, the design of the vortex treatment chamber has been modified in such a way that maximum similarity of the two chambers was ensured, i.e., the configuration above the bottom insulator as well as the outlet tubing remained unchanged. This implies that an important variable of the continuous process, the holding time of the product at T_*out*_ after the treatment chamber (Schottroff et al., 2019), remained unchanged and as low as possible. Therefore, similar additional thermal effects on the product are expected and the two chambers can directly be compared in experiments.

**FIGURE 6:**
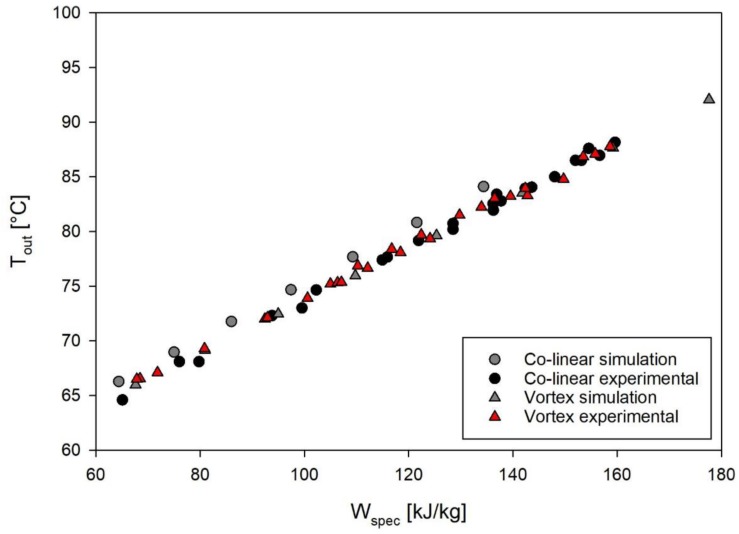
Comparison of outlet temperatures and specific energy input, as determined by experiments and simulation. Depicted experimental data points are derived from all accomplished trials with *M. lacticum* and ALP, respectively. Corresponding process parameters are given in Table 3.

In order to compare the outcomes of experiments and simulations, Figure 6 shows the measured and simulated temperatures after the treatment chambers with respect to the specific energy input. For the vortex chamber, the simulation matches the experiments well, although the temperature measurement was conducted 110 mm behind the chamber outlet. This indicates that the temperature in the pipe remains constant in good approximation and that heat transfer out of the system through the pipe wall is negligible. In case of the co-linear configuration, however, slightly higher outlet temperatures of 1–2°C above those being actually measured were determined by simulation. The deviation might be caused by experimental errors of the temperature measurement, heat transfer from the fluid to the surroundings during its passage from the treatment chamber to the temperature sensor, or slight deviations between the geometry of the chamber being used in the experiments and the simulated geometry. Since the temperatures agree fairly well between experiments and simulation under all conditions, it is expected that the CFD model allows to derive an estimate of the thermal effects of PEF on *M. lacticum* and ALP.

### Performance for Microbial Inactivation

**FIGURE 7:**
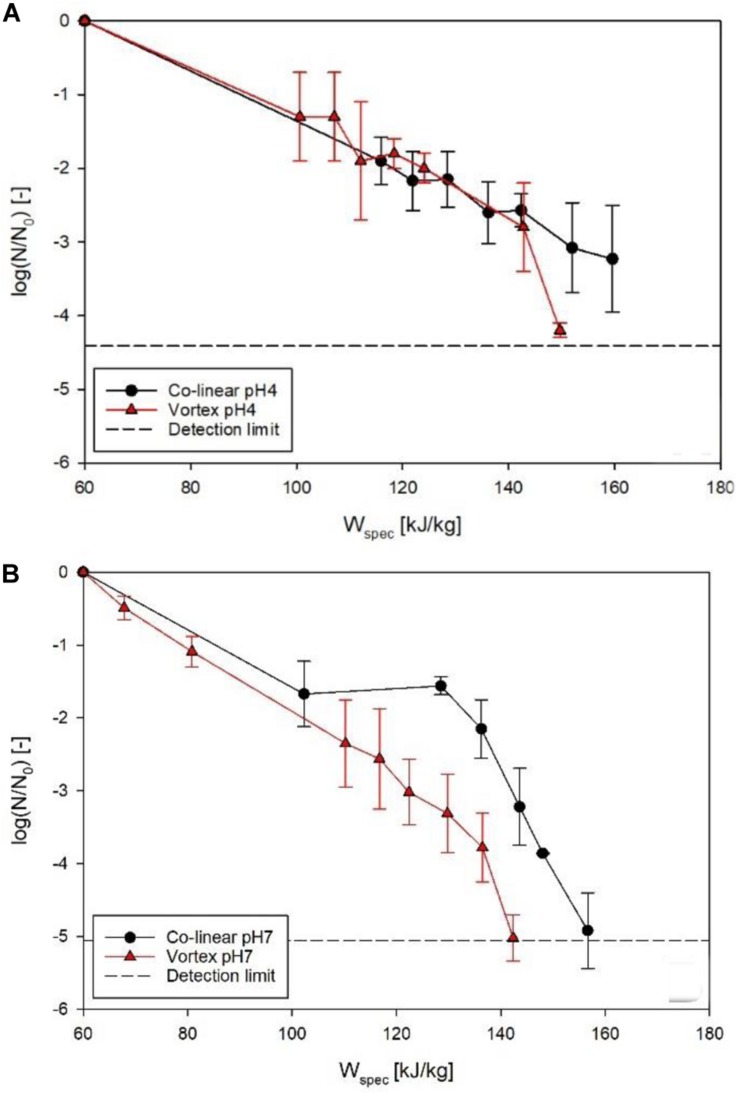
Comparison of *M. lacticum* inactivation kinetics of co-linear (black circles) and vortex (red triangles) configuration at pH 4 **(A)** and pH 7 **(B)**. Data points depict average values, error bars represent the standard deviation.

Figure 7 shows the achieved inactivation kinetics of *M. lacticum* in the co-linear and vortex PEF systems. At pH 4, the progressions of the kinetics are relatively similar, except for the highest energy input level of the vortex chamber (149.7 kJ/kg), where an additional inactivation of 1.1 log could be achieved. At pH 7, however, a distinctly increased inactivation was observed by the vortex configuration for most energy input levels, with a maximum increase of 1.8 log (143 kJ/kg).

The reason for the similar curve progression at pH 4 (Figure 7A) may be that the inactivation effect of PEF is superimposed by the effects of the low pH (García et al., 2005; Schottroff et al., 2019), which acts as an additional antimicrobial hurdle (Leistner, 1995). Therefore, even the occurrence of reversible pores in the membrane might be sufficient for an inactivation to occur, due to disturbance of the intracellular pH of the microorganism potentially associated with cell death, also at less severe processing conditions, which the cell would usually be able to survive in a more favorable environment (Toepfl, 2006). The reason for the significant increase (*p* < 0.05) in inactivation for the last kinetic point of the vortex chamber (149.7 kJ/kg) could be that for this relatively high energy input level electroporation was distinctly enhanced, exceeding the mixed effects of electric field and low pH, considering that also at pH 7 the increase in inactivation by the vortex chamber was maximized in this range of energy input levels.

In comparison, at pH 7, a significant increase (*p* < 0.05) in inactivation was shown for almost all energy input levels in the vortex configuration (Figure 7B). This leads to the conclusion that, in general, the vortex configuration produces a larger fraction of lethally injured cells, reducing the amount of sub-lethal injury. This is a beneficial attribute, as especially at neutral pH, inactivation of microorganisms by PEF may be difficult, due to the lack of additional hurdles and possible recovery of sublethally injured cells after the treatment (Schottroff et al., 2019, 2020). Moreover, the increased inactivation, especially at neutral pH, can be interpreted as a further confirmation of the increased temperature homogeneity in the vortex chamber, therefore potentially promoting possible synergistic microbial inactivation effects of temperature and electric field (Jayaram et al., 1991), by reduction of local cold spots inside the chamber.

**FIGURE 8:**
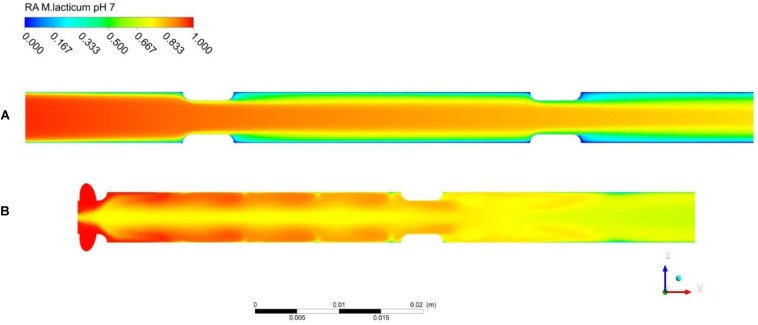
Simulated total inactivation based on residual activity (RA) of *M. lacticum* at pH 7 inside the co-linear **(A)** and vortex **(B)** treatment chamber configurations. Assumed processing conditions: $\dot{m}=7\,\mathrm{kg}/h$, *T*_*in*_ = 50°C, *E*_*avg*_ = 32*kV*/*cm*, *f*_*p*_ = 100Hz, τ_*p*_ = 3μs. Note that microbial inactivation is expressed in linear scale.

With regard to the thermal effects of PEF, Figure 8 shows contour plots of the thermal inactivation *N*/*N*_0_ (linear scale) in the co-linear and vortex chambers for a pulse repetition rate of 100 Hz and treatment conditions as defined in Table 3. In case of the co-linear chamber, it can clearly be seen that the thermal inactivation occurs in the boundary layers. The residual activity in the recirculation zone after the second insulator is even close to zero due to the long residence time and high local temperature. In contrast, the inactivation pattern in the vortex treatment chamber is distinctly different and the highest thermal inactivation can be found in the vortex core between the two treatment zones. It was observed that reverse flow toward the vortex core occurs, which causes a higher residence time of some cells in the treatment chamber, and therefore an increased thermal inactivation. However, the exposure of the product to heat seems more homogeneous in the vortex chamber due to the improved mixing.

In general, it is found that the thermal inactivation of *M. lacticum* does not take place to a significant degree and the computed values for the thermal inactivation at pH 7 are lower than 0.35 log cycles under all conditions in both treatment chambers (co-linear chamber: *RA* = 0.49 at *f*_*p*_ = 70 Hz, *RA* = 0.47 at *f*_*p*_ = 120 Hz; vortex chamber: *RA* = 0.61 at *f*_*p*_ = 70 Hz, *RA* = 0.59 at *f*_*p*_ = 120 Hz, all values in linear scale). It is also seen that the simulation of the thermal inactivation indicates a higher thermal load in the co-linear chamber in comparison to the vortex design, which becomes visible by the lower residual activity.

### Quality Effects on Heat-Sensitive Matrix Compounds

**FIGURE 9:**
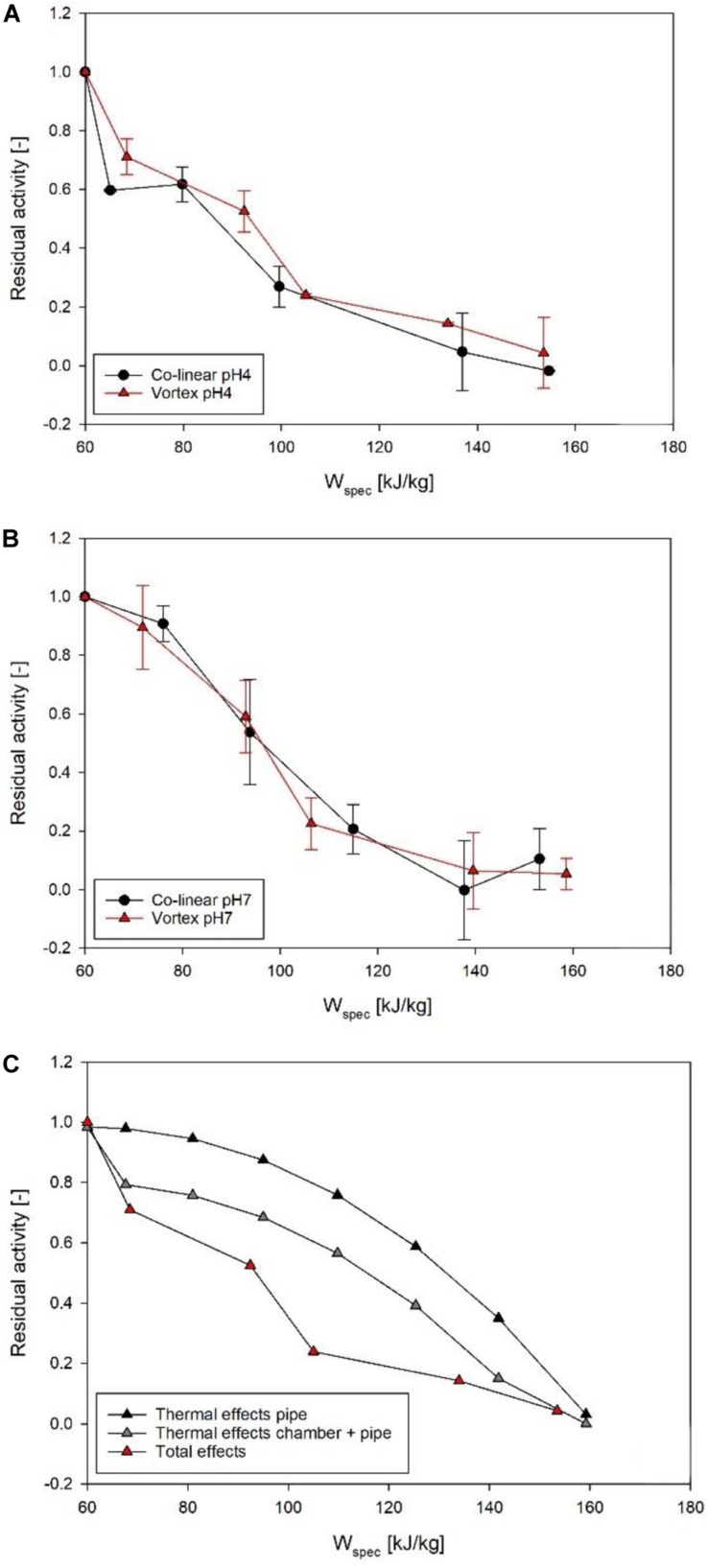
Comparison of ALP inactivation kinetics of co-linear (black circles) and vortex (red triangles) configuration at pH 4 **(A)** and pH 7 **(B)**. Data points depict average values, error bars represent the standard deviation. Further depicted **(C)** is the total inactivation in the vortex chamber at pH 4, and the thermal effects occurring in the pipe after the treatment chamber, as well as inside the treatment chamber and in the pipe, as determined by mathematical modeling and simulation.

In terms of enzyme inactivation, the effects of the individual treatment chamber designs are less obvious (Figure 9). At pH 4 (Figure 9A), the data points of the vortex chamber are located slightly above the data points of the co-linear configuration, thus indicating a minor decrease in enzyme inactivation in this treatment chamber. However, this only indicates a trend and is not statistically significant (*p* > 0.05). At pH 7, however, no difference could be determined at all, i.e., enzyme inactivation was equivalent for the co-linear and vortex chambers, independent of the respective energy input level (Figure 9B). Consequently, the quality of PEF-treated enzyme solutions by the vortex treatment chamber configuration is neither better nor worse than the quality of products processed by the conventional co-linear design. However, it has to be mentioned that the enzyme activities were determined by use of an enzyme kit, thus the precision of the measurement may be limited. Therefore, smaller variations between data points may not be detectable. In future research, more precise methods or a different product quality parameter should be deployed.

The overall measured effect of the PEF treatment on ALP can be expected to be a combination of electric field effects in the treatment chamber, thermal effects in the treatment chamber, and thermal effects in the pipe between the treatment chamber and the heat exchanger for cooling. The numerical simulation of the thermal inactivation in the vortex treatment chamber shows that the thermal inactivation explains 15–20% loss of activity (pH 4: *RA* = 0.81 at *f*_*p*_ = 70 Hz, *RA* = 0.79 at *f*_*p*_ = 120 Hz; pH 7: *RA* = 0.85 at *f*_*p*_ = 70 Hz, *RA* = 0.83 at *f*_*p*_ = 120 Hz). Although the simulation predicts steadily decreasing enzyme activities with increasing specific energy input, the differences between the individual simulations are not large enough to explain the overall inactivation observed in the experiments. For the co-linear arrangement, the simulation predicts a higher loss of ALP activity due to thermal effects. The residual activity takes values between 0.70 and 0.71, so that about 30% of the measured activity loss can be explained by thermal effects within the treatment chamber (pH 4: *RA* = 0.71 at *f*_*p*_ = 70 Hz, *RA* = 0.70 at *f*_*p*_ = 120 Hz; pH 7: *RA* = 0.70 at *f*_*p*_ = 70 Hz, *RA* = 0.70 at *f*_*p*_ = 120 Hz). Therefore, the thermal load in the vortex treatment chamber itself is considered to be lower in comparison to the co-linear design, which is an important finding in view of quality characteristics.

However, in both chambers, the decrease of enzyme activity is almost constant due to the short residence times (<1 s) and the simulation of the thermal inactivation of ALP within the respective treatment chambers could not explain the overall activity loss and its dependency on the treatment intensity as determined in the experiments (Figures 9A,B). By considering the measured temperature after the treatment chamber and the mean residence time of the enzyme between the treatment chamber and the cooling, the degree of thermal damage in the pipe can be estimated from the first order thermal inactivation kinetics of ALP as given by Eqs. 8 and 9. According to Figure 6, the measured temperature matches the simulated temperature at the outlet of the treatment chamber very well and therefore is a suitable estimate for the temperature in the pipe. The mean residence time in the pipe was determined experimentally to be 14.53 s. Figure 9C depicts the contribution of the different effects toward the loss of activity in relation to the overall effect, with respect to the specific energy input during the treatment in the vortex treatment chamber at pH 4. Similar results were obtained for the treatment at pH 7 (data not shown). It is seen that at low treatment intensities (<110 kg/kJ), the thermal load in the treatment chamber exceeds the effect in the pipe, while at higher treatment intensities the thermal damage of the enzyme in the pipe after the treatment chamber becomes the dominating feature. However, the total thermal effect still cannot explain the total measured loss of activity, which indicates that the electric field itself may have a limited effect on the enzyme activity.

It is reported that the activity of ALP may be reduced by a PEF treatment, depending on the field strength and the number of pulses (Castro et al., 2001; Poojary et al., 2017; Giteru et al., 2018). Using electroporation cuvettes, Castro et al. (2001) reported a reduction of ALP activity for field strengths as low as 13.2 kV/cm, with residual activities of 0.7 for 10 pulses, to 0.1 for 70 pulses. Temperature increase due to electric current flow was not reported. As the electric field strengths applied during the present study well surpassed these reported field strengths, non-thermal effects of PEF on ALP can be assumed. Recent studies used more controlled process conditions and reported differences between the thermal-only and the overall effects, depending on the treatment intensity. Jaeger et al. (2010) showed limited electric field effects on ALP, with around 20% increase in enzyme inactivation compared to heat alone. Electric field effects in a similar order of magnitude (up to 12%) were also reported for Lactoperoxidase (Buckow et al., 2012).

## Conclusion

The swirl flow generated in the newly designed vortex treatment chamber was shown to contribute to an increased treatment homogeneity. This could be visualized by the simulated temperature fields inside the chamber but also experimentally determined, by showing an increased microbial inactivation of *M. lacticum*, compared to the standard co-linear configuration. ALP was used as a heat-sensitive quality parameter, but the retention of this enzyme did not significantly increase. However, this could also be related to the relatively high variation of the analytical method (enzyme kit). Therefore, further research on the possible benefits of the vortex treatment chamber for quality retention should be carried out.

Nevertheless, it can be stated that hydrodynamic optimization can be a useful tool to increase PEF treatment homogeneity. During the individual trials, it was noticeable that for high energy input levels, the performance of the vortex chamber was distinctly more stable than the co-linear configuration, i.e., that arcing could be drastically reduced, which is an indication of reduced local electric field and temperature peaks. Also considering the performance for non-thermal inactivation effects on bacteria at a neutral pH, which is usually difficult to achieve (Schottroff et al., 2019), the new configuration performed comparably well. Therefore, it can be concluded that further studies using the vortex configuration and a further optimization of the system are promising, especially for the improvement of the inactivation of microorganisms by PEF at neutral pH.

Open points considering the vortex treatment chamber design are related to the further understanding of the process characteristics. Needless to say, that the design itself can be further optimized with respect to the treatment homogeneity, manufacturing or hygienic design. The design of the vortex treatment chamber was also fully based on modeling and numerical simulations, which naturally do not perfectly cover all process features. For example, in the present model the temperature dependency of some material properties was not considered in order to reduce the numerical costs of the simulation. Therefore, an additional experimental characterization of the flow and mixing in the treatment chamber should be carried out in order to validate the numerical model and to further improve its accuracy. In this regard, particle image velocimetry and particle tracking velocimetry could be suitable tools for such investigations. Open questions are also related to changes of flow and temperature distributions under different process conditions. Also, the material properties of the product, especially viscosity, will have a large impact on the process and the applicability of the new chamber design and its underlying principle of operation. For simple pipe configurations it is known that the decay of laminar swirl velocity is a function of the Reynolds number (Yao and Fang, 2012) and therefore an evaluation of the treatment conditions in terms of the Reynolds and Prandtl numbers should be part of further research on the PEF process within the vortex treatment chamber. Finally, further investigations should also address the question of upscaling. This naturally affects the flow in the treatment chamber, but also the distribution and strength of the electric field. Well-defined electric fields only exist for some simple geometric arrangements like parallel-plate configurations. However, for engineering purposes there is a need for correlations which help to estimate important parameters of the treatment (e.g., average or peak electric field strength) for different chamber designs and process scales. To date, such correlations do not exist and further studies are therefore necessary.

## Data Availability Statement

The datasets generated for this study are available on request to the corresponding author.

## Author Contributions

FS and HJ developed the idea for the study. FS, TH, and HJ were involved in the planning of study. TH, PE, JK, CM, and CR planned and conducted the modeling and simulation and analyzed the resulting data. FS and AK acquired and analyzed the experimental data. FS, JK, and CM wrote the manuscript. HJ and CR proofread the manuscript and contributed to discussions during the development of the study and the manuscript.

## Conflict of Interest

The authors declare that the research was conducted in the absence of any commercial or financial relationships that could be construed as a potential conflict of interest.
